# Phenolic Composition, Antioxidant Activity and Anti-Adipogenic Effect of Hot Water Extract from Safflower (*Carthamus tinctorius* L.) Seed

**DOI:** 10.3390/nu5124894

**Published:** 2013-11-28

**Authors:** Seok-Yeong Yu, Young-Jun Lee, Jong-Dai Kim, Suk-Nam Kang, Seong-Kap Lee, Jung-Young Jang, Hyo-Ku Lee, Jeong-Ho Lim, Ok-Hwan Lee

**Affiliations:** 1 Department of Food Science and Biotechnology, Kangwon National University, Chuncheon 200-701, Korea; E-Mails: dbtjrdud@naver.com (S.-Y.Y.); hslyj02@gmail.com (Y.-J.L.); jongdai@kangwon.ac.kr (J.-D.K.); 2 Department of Animal Resources Technology, Gyeongnam National University of Science and Technology, Jinju 660-758, Korea; E-Mail: white@gntech.ac.kr; 3 Department of Food and Biotechnology, Hoseo University, Asan 336-795, Korea; E-Mail: 2869sk@hanmail.net; 4 Department of Food Science and Technology, Kongju National University, Yesan 340-800, Korea; E-Mails: jjyoung33@hanmail.net (J.-Y.J.); hhklee@kongju.ac.kr (H.-K.L.); 5 Korea Food Research Institute, Gyeonggi 463-746, Korea; E-Mail: jhlim@kfri.re.kr

**Keywords:** antioxidant activity, phenolic compound, *Carthamus tinctorius* L. seed, 3T3-L1 adipocyte, hot-water extract

## Abstract

This study was to evaluate the phenolic content and composition of *Carthamus tinctorius* L. seed extract (CSE) and to further assess its antioxidant and anti-adipogenic activities using various radical scavenging systems and 3T3-L1 cells. Our results show that the total phenolic and flavonoid contents of CSE were 126.0 ± 2.4 mg GAE/g and 62.2 ± 1.9 mg QE/g, respectively. The major phenolic compounds in CSE was (−)-epigallocatechin (109.62 mg/g), with a 4-hydroxy benzhydrazide derivative and gallocatechin present at 18.28 mg/g and 17.02 mg/g, respectively. CSE exhibited remarkable radical scavenging activities, FRAP (ferric reducing antioxidant power) and reducing power in a dose-dependent manner. Moreover, the oxygen radical absorbance capacity (ORAC) value of CSE (0.1 mg/mL) was 62.9 ± 4.7 μM TE (trolox equivalent)/g. During adipogenesis, CSE significantly inhibited fat accumulation in 3T3-L1 cells compared with control cells. Overall, these results indicate that CSE might be a valuable source of bioactive compounds that impart functional food and natural antioxidant properties.

## 1. Introduction

Recently, phytochemicals and nutraceuticals have been used to prevent the formation of free radicals and ROS (reactive oxygen species). ROS are generated by aerobic respiration as well as various environmental stress factors, including pollution, temperature, and nutritional limitation [1]. As ROS trigger numerous deleterious effects, such as cancer, cell injury, and muscular degeneration, interest in plants as exogenous antioxidants has been increased [2,3,4]. Accordingly, phenolic compounds such as flavonoids and anthocyanins are becoming recognized as bioactive compounds for defense ROS, and their efficacy has indeed been reported [5,6].

The *Carthamus tinctorius* L. seed is a traditional herbal medicine and has been used across Asia, including in Korea. *Carthamus tinctorius* L. seeds are known to be a plentiful source of α-linoleic acid and have been used for cooking oil and vegetable oil in Europe. Regarding the efficacy, *Carthamus tinctorius* L. seeds have protective effects on osteoporosis and rheumatoid arthritis, as well as a beneficial effect on atherogenic risk [7,8,9,10]. Recently, it was discovered that *Carthamus tinctorius* L. seeds contain various phenolic compounds, such as lignin and flavonoids [11,12]. Moreover, the anti-atherogenic, antioxidative, and antimelanogenic activities of these compounds have been discovered by previous studies [13,14,15]. To foster these effects, *Carthamus tinctorius* L. seeds have been used by Koreans as a type of hot water extract, and the quality of the drinks and tea bags processed with *Carthamus tinctorius* L. seeds has recently been evaluated [16]. Moreover, it has been reported that various flavonoids and phenolic acids have anti-obesity activity and induce apoptosis in 3T3-L1 preadipocytes, and that ethyl acetate extract from *Carthamus tinctorius* L. seeds ameliorate the blood status in estrogen-deficient rats [17,18,19]. However, investigations regarding the phenolic compounds in hot water extracts, including tea bag products containing *Carthamus tinctorius* L. seeds, are still lacking, even though the extract and tea are used for herbal medicine. In addition, it remains unclear whether the hot water extract from *Carthamus tinctorius* L. seeds has beneficial effects on obesity and desirable antioxidant activity against ROS.

Therefore, the aim of this study was to investigate the phenolic composition, total phenolic and flavonoids contents, antioxidant activity and anti-adipogenic effect of *Carthamus tinctorius* L. seed extract (CSE) to determine the usefulness of CSE as a functional food ingredient and natural antioxidant.

## 2. Methods and Materials

### 2.1. Materials

*N*-Acetyl-l-cysteine (NAC), Oil Red O (ORO), 6-hydroxy-2,5,7,8-tetramethylchroman-2-carboxylic acid (trolox), 3-isobutyl-1-methylxanthine (IBMX), insulin, dexamethasone (DEX), 1,1-diphenyl-2-picrylhydrazyl (DPPH), acetic acid, gallic acid, 2,2′-azino-bis(3-ethylbenzothiazoline-6-sulfonic acid) diammonium salt (ABTS), isopropanol, Folin & Ciocalteu’s phenol reagent, sodium carbonate, 2,4,6-tris(2-pyridyl)-s-triazine (TPTZ), trichloroacetic acid (TCA), potassium ferricyanide, and potassium persulfate were procured from Sigma (St. Louis, MO, USA), Dulbecco’s modified Eagle’s medium (DMEM), bovine serum (BS), fetal bovine serum (FBS), penicillin-streptomycin (P/S), phosphate-buffered saline (PBS), and trypsin-EDTA were obtained from Gibco (Gaithersburg, MD, USA).

### 2.2. Sample Preparation

*Carthamus tinctorius* L. seeds were purchased from the Farming Corporation in the oriental medicine area of Jecheon, South Korea. Cleaned *Carthamus tinctorius* L. seeds were ground using a grinder (IKA M20, IKA, Staufen, Germany). The *Carthamus tinctorius* L. seed powder was refluxed with 10 volumes (v/w) of hot water at 80 °C for 3 h, and the CSE was filtered through Whatman No. 3 filter paper, with each filtration repeated two times. The filtrate was concentrated with a rotary evaporator (N-1000SWD, Eyela, Tokyo, Japan) at 55 °C and then freeze dried using a freeze dryer (Biotron, Gangneung, Korea). Freeze-dried powder was stored at −20 °C until use for further study.

### 2.3. Determination of Total Phenolic and Flavonoid Contents

The total phenolic content of CSE was determined as described by Gutfinger [20]. To 1 mL of the sample, 1 mL of 10% (v/v) Folin & Ciocalteau’s reagent and 1 mL of 2% (w/v) Na_2_CO_3_ were added, and the mixture was incubated at 25 °C for 1 h. The reactant absorbance was measured at 750 nm. The calibration curve (*y* = 6.1377*x* + 0.0103) was calculated using gallic acid and the results were expressed in mg of gallic acid equivalent (GAE)/g.

The total flavonoid content was determined by the method of Moreno *et al*. [21]. To 0.5 mL of the sample in a test tube, 0.1 mL of aluminum nitrate (10%, w/v), 0.1 mL of 1 M potassium acetate, and 94% (v/v) ethanol were added and the mixture was incubated at 25 °C for 40 min. The reactant absorbance was measured at 415 nm. The calibration curve (*y* = 3.3533*x* + 0.0377) was calculated using quercetin and the results were expressed in mg of quercetin equivalent (QE)/g.

### 2.4. HPLC Analysis of Phenolic Compounds in CSE

CSE was dissolved in DMSO and filtered with a Millipore membrane filter (0.45 μm) prior to HPLC analysis. Analysis of the phenolic compounds was conducted using an Agilent 1100 series HPLC equipped with a diode array detector (DAD). The dissolved sample was separated on a Nucleosil 100-5 C_18_ column (250 mm × 4.0 mm i.d., 5 μm particle size). The mobile phase was composed of two solvents: solvent A was a mixture of water/formic acid (pH 3.29), and solvent B was 100% acetonitrile/formic acid (pH 3.29). An elution gradient was performed as follows: initially 7.0% of solvent B, followed by 0% to 15% B over 25 min, 30% B at 35 min, 40% B at 50 min, 100% B at 45 min, 100% B at 55min, 50% B at 60 min, 50% B at 63 min, 0% B at 64 min, and 0% B at 73 min. The flow rate was 1.0 mL/min, and the column temperature was 30 °C.

The DAD acquisitions were integrated at 280 nm (for gallic acid, 4-hydroxy benzhydrazide, epigallocatechin, epicatechin, epigallocatechin gallate, vanillic acid, (+)-catechin hydrate, syringic acid, phloroglucinol, catechin gallate, gallocatechin, protocatechuic acid, *p*-anisic acid, and 3-(3,5-dimethyl-pyrazol-1-yl) benzoic acid), 320 nm (for chlorogenic acid, caffeic acid, *p*-coumaric acid, trans-ferulic acid, naringin, 2-amino-3,4-dimethyl-benzoic acid, coumarin, morin hydrate, luteolin, hesperetin, alizarin, biotin, and trans-chalcone), and 370 nm (for rutin hydrate, myricetin, quercetin hydrate, quercetin dihydrate, rhein, 3-hydroxyflavone, kaempferol, and emodin). The identification of the phenolic acids and flavonoids was achieved by comparing retention times.

### 2.5. Determination of *in Vitro* Antioxidant Capacity

The 2,2-diphenyl-1-picrylhydrazyl (DPPH) assay was performed according to the method of Kim *et al*. [22] with some modification. A solution of 0.4 mM DPPH in anhydrous ethanol was stirred for 30 min, and the absorbance of the solution was adjusted to 1.1 ± 0.1 at 490 nm. Then, 0.2 mL of sample or control were mixed with 0.8 mL of DPPH solution and incubated for 4 min in the dark. The decrease in absorbance was monitored at 490 nm. The control consisted of 0.2 mL of distilled water in 0.8 mL of DPPH solution.

The 2,2′-azino-bis(3-ethylbenzthiazoline-6-sulphonic acid) (ABTS) assay was based on the method of Roberta *et al*. [23], with some modifications. Briefly, 7 mM of ABTS was mixed with 2.45 mM of potassium persulfate, and the ABTS-potassium persulfate (1:0.5, v/v) was incubated at 20 °C for 16 h. The blue-green ABTS+ solution was diluted with 95% (v/v) ethanol until the absorbance reached 0.70 ± 0.02 at 734 nm. Then, 10 μL of the sample was mixed with 1 mL of ABTS+ solution. The absorbance of the mixture was monitored at 734 nm after 6 min. For the blank, 10 μL of water instead of the sample was used and each sample was measured in triplicate.

The Ferric reducing antioxidant power (FRAP) assay was conducted using the method described by Benzine and Strain [24] with some modifications. The FRAP reagent consisted of 0.3 M sodium acetate buffer (pH 3.6), 10 mM 2,4,6-tripyridyl-s-triazine (TPTZ) and 20 mM FeCl_3_∙6H_2_O at a ratio of 10:10:1 (v/v). The sample (50 μL) was mixed with 150 μL of distilled water and 1.5 mL of the reagent, and the mixture was incubated at 37 °C for 4 min. The absorbance of the mixture was monitored at 593 nm.

The reducing power assay was performed according to the method of Yen and Duh [25]. In brief, 0.5 mL of the sample was mixed with 2.5 mL of sodium phosphate buffer (pH 6.6) and 2.5 mL of potassium ferricyanide (1%, v/v). The mixture was incubated at 50 °C for 20 min. Then, to the reactant, 2.5 mL of trichloroacetic acid (10%, w/v) was added and the mixture was centrifuged at 1790× *g* for 10 min. A 2.5 mL aliquot of the supernatant was mixed with 2.5 mL of double distilled water and 0.5 mL of potassium chloride (0.1%, w/v), and the absorbance of the mixture was monitored at 700 nm.

For the determination of the correlation coefficient among *in vitro* antioxidant capacity assays, the *R* squared (*R*^2^) value of the linear equation was used. In brief, the results of each model were arranged in order of increasing concentration, and the linear equation between two models was defined. Then, the *R*^2^ of the linear equation was measured. 

The ORAC assay was based on the method of Ou *et al*. [26] and was carried out on a COBAS FARA II spectrofluorometer with a centrifugal analyzer (Roche Diagnostic System Inc., Branchburg, NJ, USA). The experiment was conducted in 75 mM phosphate buffer (pH 7.4) at 37 °C. The sample (25 μL) and 150 μL of 40 nM fluorescein (FL) were placed in each well of a microplate. Then, 25 μL of 150 mM AAPH was added, and the plate was immediately placed in the reader. The reader was programmed to record the fluorescence of FL every two minutes for 60 min at emission and excitation wavelengths of 535 nm and 485 nm, respectively. Trolox, a water-soluble analog of vitamin E, was used as a standard, and phosphate buffer instead of the sample was measured for a blank. The results were expressed as μmole trolox equivalent/g, and at least three independent assays were performed for each sample.

### 2.6. Cell Culture

The 3T3-L1 adipocytes, provided American Type Culture Collection (ATCC, CL-173, Manassas, VA, USA), were cultured and differentiated into adipocytes according to the method of Blumberg *et al*. [27]. The 3T3-L1 cells were cultured in DMEM containing 10% (v/v) BS and penicillin in a humidified atmosphere of 95% air and 5% CO_2_ at 37 °C. The medium was replaced every 2 days. The 3T3-L1 cells were trypsinized and reseeded in 24-well plates before they reached confluency. After they reached confluence, the cells were maintained for 2 days. Then, preadipocytes were induced to differentiate into adipocytes by DMEM supplemented with 10% (v/v) FBS, 10 mg/mL insulin, 1 mM IBMX, and 0.1 mM DEX. Two days later, the medium was exchanged to DMEM supplemented with 10% FBS and 10 mg/mL insulin, and refreshed every other day.

### 2.7. Determination of Lipid Accumulation by Oil Red O Staining

To determine the lipid accumulation, the 3T3-L1 cells were stained with Oil Red O as described Blumberg *et al*. [27] with slight modifications. After 8 days, the cells were washed with phosphate-buffered saline (pH 7.4) twice and fixed to the plates with 10% formaldehyde for 1 h. Following fixation, each well was washed with 60% isopropanol and completely dried. An Oil Red O stock solution was made by combining 2 g of Oil Red O powder with 100 mL of an isopropanol and distilled water mixture at a ratio of 60:40 (v/v). Then, the cells were stained with the Oil Red O stock solution for 15 min at room temperature, washed four times with distilled water, and dried. The red dye retained in the cells was eluted into isopropanol and quantitated with a microplate reader (Molecular Devices, Sunnyvale, CA, USA) at 490 nm.

### 2.8. Statistical Analysis

All experiments were repeated three times. Data are presented as the means ± standard deviation. The data were analyzed by one-way analysis of variance (ANOVA), and all the grouped data were evaluated by one-way analysis of variance followed by the least significant difference test using the SAS package (SAS Institute, Cary, NC, USA) version 9.2. Values with *p* < 0.05 were considered significantly different between the treatments.

## 3. Results

### 3.1. Total Phenolic and Flavonoid Contents of CSE and Its Phenolic Compounds

The total phenolic and flavonoid contents of CSE were 126.0 ± 2.4 mg GAE/g and 62.2 ± 1.9 mg QE/g, respectively (Table 1). The phenolic compounds of the *Carthamus tinctorius* L. seed were analyzed by HPLC. 

**Table 1:** Total phenolic and total flavonoid contents of *Carthamus tinctorius* L. seed extract (CSE).

	Total Phenolic Content (mg GAE ^1^/g)	Total Flavonoid Content (mg QE ^2^/g)
CSE ^3^	126.0 ± 2.4 ^4^	62.2 ± 1.9

^1^ GAE: gallic acid equivalent; ^2^ QE: quercetin equivalent; ^3^ CSE: *Carthamus tinctorius* L. seed extract; ^4^ Means ± S.D. in triplicate (*p* < 0.05).

As summarized in Table 2, (−)-epigallocatechin (109.6 mg/g) was the highest, and the ester of (−)-epigallocatechin was 1.1 mg/g. In the case of the phenolic acids, the 4-hydroxybenzhydrazide derivative and 2-amino-3,4-dimethylbenzoic acid were 18.2 and 16.8 mg/g, and chlorogenic acid, syringic acid, *p*-coumaric acid, and trans-ferulic acid were 2.4, 0.2, 0.5, and 3.0 mg/g, respectively. The *Carthamus tinctorius* L. seed also contained trans-chalcone (2.1 mg/g), as well as flavonoids, including gallocatechin (17.0 mg/g), quercetin dehydrate (2.2 mg/g), kaempferol (0.8 mg/g), rutin hydrate (3.7 mg/g), luteolin (1.6 mg/g), and naringin (6.0 mg/g). In the last ten years, many phenolic compounds in the *Carthamus tinctorius* L. seed have been isolated, and these compounds have become associated with antioxidant activity. The phenolic compounds isolated from the *Carthamus tinctorius* L. seed were lignin, serotonin derivatives and serotonin glycosides, such as, *N*-(*p*-coumaroyl) serotonin, 8′-hydroxyarcgenin-4′-*Ο*-β-d-glucoside, *N*-feruloylserotonin, *N*-(*p*-coumaroyl) serotonin-5-*O*-β-d-glucoside, *N*-feruloylserotonin-5-*O*-β-d-glucoside [12,28].

**Table 2:** Phenolic compounds in *Carthamus tinctorius* L. seed extract (CSE).

Phenolic Compounds	mg/g, Dry Base
4-Hydroxybenzhydrazide derivative	18.2
2-Amino-3,4-dimethylbenzoic acid	16.8
Chlorogenic acid	2.4
Syringic acid	0.2
*p*-Coumaric acid	0.5
*trans*-Ferulic acid	3.0
Gallocatechin	17.0
(−)-Epigallocatechin	109.6
Epigallocatechin gallate	1.1
Quercetin dehydrate	2.2
Kaempferol	0.8
Rutin hydrate	3.7
Luteolin	1.6
Naringin	6.0
*trans*-Chalcone	2.1

### 3.2. Effects of CSE on *in Vitro* Antioxidant Capacity

**Figure 1:**
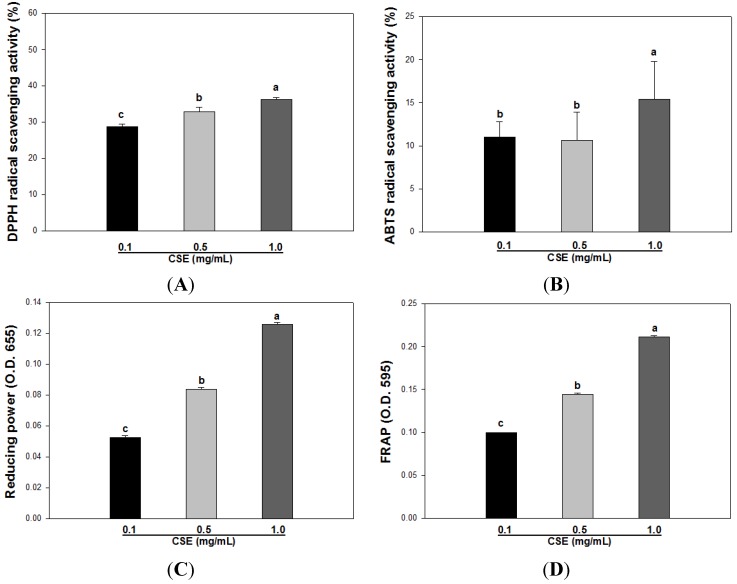
DPPH radical scavenging activity (**A**), ABTS radical scavenging activity (**B**), reducing power (**C**), and FRAP value (**D**) of *Carthamus tinctorius* L. seed extract (CSE). Each bar indicates the mean ± S.D. in triplicate. Statistical analysis was performed by one-way ANOVA (*p* < 0.05).

At concentrations of 0.1, 0.5, and 1.0 mg/mL, the antioxidant capacity of CSE was evaluated by various *in vitro* models, such as DPPH radical scavenging, ABTS radical scavenging, the FRAP assay, and the reducing power assay. As shown Figure 1A, at concentrations of 0.1, 0.5, and 1.0 mg/mL, the DPPH radical scavenging activity of CSE was 28.7 ± 0.7, 32.9 ± 1.1, and 36.2% ± 0.5%, respectively. The ABTS radical scavenging activity of CSE was 11.0 ± 1.7, 10.6 ± 3.2, and 15.3 ± 4.4, respectively (Figure 1B). Moreover, Figure 1C,D shows the reducing power and the FRAP of CSE. The reducing power of CSE was 0.052, 0.085, and 0.126, respectively, and in the FRAP assay, the absorbance of each concentration was 0.099, 0.144, and 0.211, respectively. 

Table 3 shows the correlation coefficient among *in vitro* antioxidant capacity assays. The correlation coefficient was highest (*R*^2^ = 1.000) between the FRAP assay and the reducing power assay. On the other hand, the correlation coefficient between ABTS and DPPH radical scavenging activity was lowest (*R*^2^ = 0.6164). For the DPPH assay, correlation coefficients with the FRAP (*R*^2^ = 0.9672) assay and the reducing power assay (*R*^2^ = 0.9687) were observed. The correlation coefficients of the ABTS with the FRAP and reducing power assays were 0.7820 and 0.7785, respectively.

**Table 3:** Correlation coefficients (*R*^2^) between 2,2-diphenyl-1-picrylhydrazyl (DPPH), 2,2′-azino-bis(3-ethylbenzothiazoline-6-sulphonic acid) (ABTS), Ferric reducing antioxidant power (FRAP), and the reducing power of *Carthamus tinctorius* L. seed extract (CSE).

	DPPH	ABTS	Reducing Power	FRAP
DPPH	-	0.6139 **	0.9752 **	0.9679 **
ABTS	0.6139 **	-	0.7597 **	0.7782 **
Reducing power	0.9752 **	0.7597 **	-	0.9995 *
FRAP	0.9679 **	0.7782 **	0.9995 *	-

* Correlation is significant at *p* < 0.05; ** Correlation is significant at *p* < 0.01.

**Figure 2:**
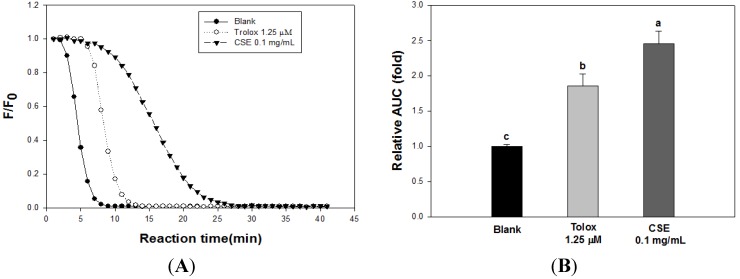
Effect of *Carthamus tinctorius* L. seed extract (CSE) on fluorescein decay induced by AAPH. (**A**) Decay curve of fluorescence in the presence or absence of samples, CSE 0.1 mg/mL (▼), Trolox 1.25 μM TE/g (◯), and Blank (●). (**B**) Radical scavenging activity of CSE was expressed as a value relative to blank. Each bar indicates the mean ± S.D. in triplicate. The decay curve was determined by fluorescein fluorescence intensity (excitation at 493 nm, emission at 515 nm).

The ORAC value of CSE is illustrated in Figure 2. Figure 2A presents the decay curve of fluorescein with or without CSE. Figure 2B shows the ORAC value of CSE at a concentration of 0.1 mg/mL, which was 62.9 ± 4.7 μM TE/g.

### 3.3. Cell Viability of CSE in 3T3-L1 Preadipocytes

**Figure 3:**
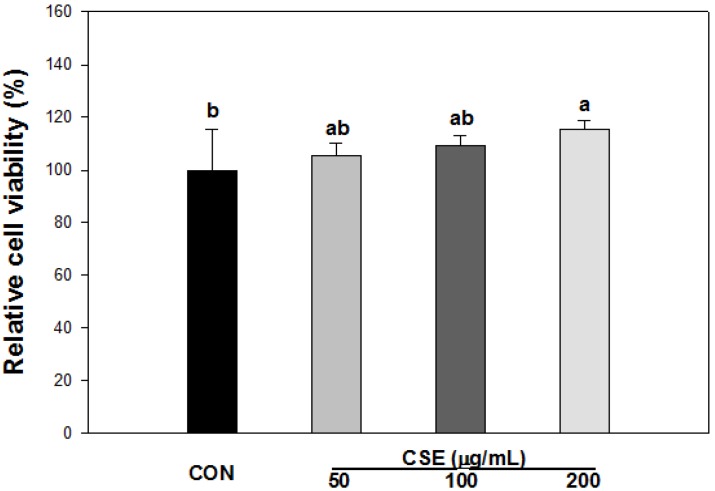
Effect of *Carthamus tinctorius* L. seed extract (CSE) on cell viability. XTT assay was used for cell viability. 3T3-L1 cells were seeded in a 96-well plate, and then incubated with CSE at the indicated concentrations for 24 h. After incubation, the cells were exposed to XTT reagent containing PMS for 4 h, and then were subjected to the analysis of cell viability. Each value is the mean ± S.D. of the results from six different plates (*n* = 6) and is representative of the results from at least three different experiments. Statistical analysis was performed by one-way ANOVA (*p* < 0.05). CON: Control, CSE: *Carthamus tinctorius* L. seed extract.

XTT was used to detect the cell viability of CSE on 3T3-L1 cells. According to the results, different concentrations of CSE had no significant inhibitory effects on 3T3-L1 preadipocyte proliferation after a 24 h incubation period (Figure 3). When the absorbance values obtained in the culture media without CSE were expressed as 100% ± 15.3%, the absorbance values obtained after CSE treatments of 50, 100, and 200 μg/mL were 105 ± 4.5, 109 ± 3.8, and 115% ± 3.4% after 24 h of incubation, respectively. CSE has no cytotoxicity for a concentration range up to 200 μg/mL and was used for the anti-adipogenic activity test.

**Figure 4:**
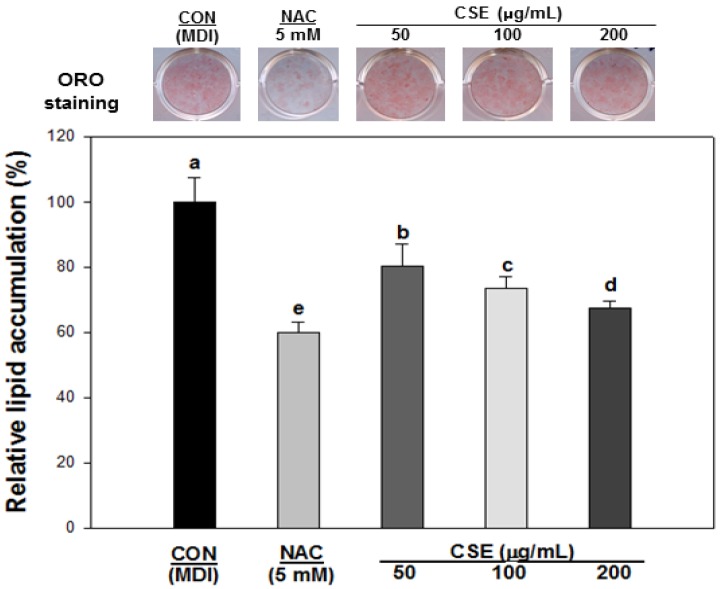
Effect of *Carthamus tinctorius* L. seed extract (CSE) on adipogenesis during the differentiation of 3T3-L1 cells. Oil red O staining at day 8 in presence or absence of CSE. Lipid accumulation was determined by absorbance at 490 nm (Abbreviations: CON: Control, NAC: *N*-Acetyl cysteine, CSE: *Carthamus tinctorius* L. seed extract). Each value is the mean ± S.D. in triplicate. Bars with different letters indicate statistically significant differences among groups at *p* < 0.05 by one-way ANOVA.

### 3.4. Effect of CSE on Lipid Accumulation in 3T3-L1 Cells

We assessed the suppressive effects of CSE on adipocyte differentiation using Oil Red O staining during the differentiation of 3T3-L1 preadipocytes. The cells were incubated in the presence or absence of CSE (50, 100, and 200 μg/mL) for 8 days, and 5 mM NAC was used as a positive control. The lipid accumulation in 3T3-L1 cells is shown in Figure 4. When preadipocytes differentiated into adipocytes, the lipid accumulation in cells treated with CSE was markedly inhibited in a dose-dependent manner.

## 4. Discussion

The *Carthamus tinctorius* L. seed has been used in traditional medicine in Asia including in Korea. It has also been used for osteoporosis and arthritis therapy in Korea [29]. Lignin, flavonoids, and serotonin derivatives have been isolated from *Carthamus tinctorius* L. seed [11]. These compounds have been investigated for various physiological activities such as anti-cancer, antioxidant and anti-inflammatory effects [8,30,31]. Recently, *Carthamus tinctorius* L. seeds have received considerable attention in Korea, as *Carthamus tinctorius* L. seeds are known to have various physiological functions and they are used as an ingredient in medicinal herbal tea. Unfortunately, the antioxidant activity of hot water extract from *Carthamus tinctorius* L. seeds was poorly evaluated and also was not measured using various *in vitro* models, such as DPPH radical scavenging activity, ABTS radical scavenging activity, FRAP and reducing power as well as ORAC assay. In addition, the correlation between the antioxidant activity of *Carthamus tinctorius* L. seeds and adipogenesis is unclear but it was reported that lipid accumulation in 3T3-L1 adipocytes coincides with the increase in oxidative stress [32]. Therefore, we investigated the phenolic compounds and physiological activities of *Carthamus tinctorius* L. seeds by various *in vitro* antioxidant models and screened the correlation between the antioxidant activity of *Carthamus tinctorius* L. seed and anti-adipogenesis in 3T3-L1 adipocytes.

Many phenolic compounds such as serotonin derivatives, serotonin glycosides, lignin and flavonoids were isolated from *Carthamus tinctorius* L. seeds, and these compounds comprise a large proportion of the phenolic compounds [11]. As mentioned previously, the phenolic content of CSE in the present study was 127.0 ± 2.4 mg GAE/g. The total flavonoid content of CSE was 62.2 ± 1.9 mg QE/g. In addition, the phenolic compounds from the *Carthamus tinctorius* L. seeds were analyzed by HPLC. The major phenolic compound in *Carthamus tinctorius* L. seeds was (−)-epigallocatechin, which had a high content (109.6 mg/g). Moreover, several flavonoid compounds, such as kaempferol, rutin hydrate, luteolin, baringin, and quercetin hydrate, were detected by HPLC. According to previous studies, various phenolic compounds, such as 8′-hydroxyarctigenin-4′-*O*-β-d-glucoside (HAG), matairesinol, n-feruloylserotonin 5-*O*-β-d-glucoside, *N*-(*p*-coumaroyl) serotonin), and acacetin, are present in *Carthamus tinctorious* L. seeds, and serotonin derivatives were found to be the major compound in the *Carthamus tinctorious* L. seed [11,28]. However, we found that *Carthamus tinctorious* L. seeds consist of various flavonoids, as described in Table 2, apart from acacetin and serotonin derivatives, and we expect that these phenolic compounds contribute to enhancing the bioactivity of the *Carthamus tinctorious* L. seed.

Antioxidative activities are determined by various *in vitro* assays including DPPH radical scavenging activity, ABTS radical scavenging activity and FRAP, which are typically utilized and convenient in their application. However, these assays are based on a particular mechanism, such as scavenging free radicals and affecting reducing power. Therefore, various assays are required to determine the antioxidant activity of *Carthamus tinctorius* L. seeds, and we used diverse methods, such as DPPH, ABTS, FRAP, reducing power, and oxygen radical absorbance capacity (ORAC) [33,34]. As shown in Figure 1, the antioxidant activities of CSE were increased in a dose-dependent manner, and we found that CSE has the ability to scavenge radicals and protect oxidation. In addition, we measured the correlation coefficients between the assays, which are summarized in Table 3. The present study showed that the FRAP and reducing power assays were more strongly correlated with the ABTS assay than with the DPPH assay. The FRAP and reducing power assays have the strongest correlation because of their concomitant mechanisms. However, the correlation coefficient between the DPPH and ABTS assays was the lowest. A correlation between the DPPH and ABTS assays was reported in previous studies on fruits [34,35]. In fruits, the correlation coefficient from the aforementioned studies showed a strong positive correlation between the assays, but the correlation was lower in vegetables. Therefore, we found that the *Carthamus tinctorius* L. seed is more similar to vegetables regarding the correlation between these assays. Moreover, as shown Figure 2A, the ORAC assay of CSE was higher than that of trolox (1.25 μM), and the value was 62.9 ± 4.7 μM TE/g. Boxin *et al*. [36] established the ORAC values of various vegetables, such as pea, carrot, white cabbage, tomato, snap bean, white onion, red pepper, cauliflower, beet, broccoli, purple onion, spinach, and green pepper. The highest value for vegetables was found in green pepper (160 μM TE/g) and the lowest value was found for pea (18 μM TE/g). When compared with the vegetables above on ORAC value, our ORAC value for CSE is similar to the ORAC value (66 μM TE/g) of tomato.

The anti-adipogenic effect of the *Carthamus tinctorius* L. seed was evaluated through lipid accumulation in the 3T3-L1 adipocytes. After the differentiation of preadipocytes into adipocytes, a significant number of lipid droplets accumulated in the intracellular membrane [37]. In addition, Furukawa *et al*. [32] revealed that lipid droplets accumulated in the white adipocyte tissue (WAT), with the NADPH oxidase gene and the ROS augmented during the differentiation. Based on this procedure, we treated 3T3-L1 adipocytes with CSE. As shown in Figure 4, CSE-treated adipocytes suppressed lipid accumulation in a dose-dependent manner. Given the effect constraining lipid accumulation, the efficacy against ROS production should be evaluated, and then the mechanism of its efficacy will be able to elucidate by signaling pathway involved in ROS production and adipogenesis.

We suggest that the hot water extract obtained from *Carthamus tinctorius* L. seeds can scavenge free radicals and prevent oxidation in various *in vitro* antioxidant models as well as reduce the lipid accumulation in 3T3-L1 cells.

## 5. Conclusions

Through various experiments, CSE presented various phenolic compounds and exhibited the ability to help regulate the redox system as well as suppress lipid accumulation in 3T3-L1 adipocytes. Therefore, these results indicate that *Carthamus tinctorius* L. seeds might be a potent natural resource that can be used in various applications, such as in therapy and in functional foods. We suggest that further studies based on this study are needed to elucidate the effect of CSE on anti-oxidative activity as well as its effect on adipogenesis
